# Predictors of treatment outcome in higher levels of care among a large sample of adolescents with heterogeneous eating disorders

**DOI:** 10.1186/s13034-024-00819-8

**Published:** 2024-10-17

**Authors:** Erin E. Reilly, Sasha Gorrell, Alan Duffy, Dan V. Blalock, Philip Mehler, Harry Brandt, Susan McClanahan, Kianna Zucker, Naomi Lynch, Simar Singh, Catherine R. Drury, Daniel Le Grange, Renee D. Rienecke

**Affiliations:** 1 Department of Psychiatry and Behavioral Sciences, University of California, San Francisco, San Francisco, CA USA; 2 Eating Recovery Center/Pathlight Mood & Anxiety Center, Denver, CO USA; 3 Center of Innovation to Accelerate Discovery and Practice Transformation, Durham Veterans Affairs Medical Center, Durham, NC USA; 4 Department of Psychiatry and Behavioral Sciences, Duke University Medical Center, Durham, NC USA; 5 Department of Psychiatry & Behavioral Neuroscience, The University of Chicago (Emeritus), Chicago, IL USA; 6 Acute Center for Eating Disorders at Denver Health, Denver, CO USA; 7 University of Colorado School of Medicine, Denver, CO USA; 8 Department of Psychiatry and Behavioral Sciences, Northwestern University, Chicago, IL USA

**Keywords:** Adolescents, Eating disorders, Treatment, Outcomes, Higher levels of care

## Abstract

**Background:**

Despite widespread use of higher levels of care in treating eating disorders in adolescents, research supporting the use of these treatments remains limited by small sample sizes and a predominant focus on anorexia nervosa. Further, existing data regarding predictors of outcome have yielded mixed findings. In the current study, we evaluated treatment outcomes and predictors of outcome among a large sample of adolescents with eating disorders presenting to inpatient, residential, partial hospitalization programs, and intensive outpatient programs across the United States.

**Methods:**

Adolescents (*N* = 1,971) completed self-report measures of eating disorder symptoms, depression, and anxiety at treatment admission, stepdown, and discharge. Using linear mixed effect models, we evaluated changes in symptoms over treatment separately among youth admitted to inpatient/residential treatment and those admitted to partial hospitalization/intensive outpatient programs, and used established metrics to gauge frequency of reliable (i.e., statistically reliable) and clinically significant change.

**Results:**

Results suggested decreases in eating disorder symptoms, depression, and anxiety from intake to discharge. Around 50% of the sample reported reliable decreases in eating disorder symptoms at stepdown and discharge, with 30% of the sample reporting reliable reductions in depression and anxiety. Psychiatric comorbidity, primary diagnosis, age, and eating disorder symptoms at admission consistently predicted treatment-related change, although patterns in findings varied across symptoms.

**Conclusions:**

Data from our sample are consistent with past work suggesting that adolescents enrolled in higher levels of care report clinical benefit; however, these effects are heterogenous, and a significant portion of individuals may not report reliable change in symptoms. Ultimately, ongoing work is required to better understand how and for whom higher levels of care may achieve their benefit and to identify the optimal approach for improving outcomes for adolescents with eating disorders.

**Supplementary Information:**

The online version contains supplementary material available at 10.1186/s13034-024-00819-8.

## Background

Eating disorders (EDs), such as anorexia nervosa (AN), bulimia nervosa (BN), and binge eating disorder (BED), commonly onset in adolescence [1] and are associated with significant functional impairment, severe medical complications, decreased quality of life, and increased mortality risk [2]. Across diagnostic groups, EDs are often associated with a chronic course [3] and duration of untreated illness predicts poorer outcome [4], highlighting a critical need to prioritize improvements in interventions.

Higher levels of care (HLOCs) (including intensive outpatient program (IOP), partial hospitalization program (PHP), residential (RES), and inpatient (IP) psychiatric and medical stabilization treatment programs) are commonly needed for the treatment of individuals with EDs, particularly individuals who present with severe ED-specific symptoms and/or medical instability, or who do not have access to local care options [5]. Further, the past decades have seen increased interest in exploring stepped care and/or staging models, wherein individuals are initially assigned to an optimal level of care matched with their clinical presentation, then stepped down to progressively lower levels of care [6–8]. Although there are some consensus guidelines to inform decisions related to intensity of care when a patient with an ED presents for treatment [9–11], improvements in empirically driven approaches to clinical decision making are needed in order to improve the prognosis for young people with EDs. Accordingly, data exploring both the effectiveness of HLOCs for EDs as well as factors associated with treatment response will be useful in informing personalized intervention.

To date, effectiveness data for different HLOCs remain limited, particularly considering the frequency of use of these interventions and their high cost, which may inhibit help-seeking behaviors and/or access to care [5, 12, 13]. Existing research indicates overall benefits of HLOCs for decreasing both ED-specific and other co-occurring symptoms (e.g., anxiety) [14–26]. However, existing studies also suggest high levels of heterogeneity in response, as well as low rates of long-term follow-up [14, 27, 28]. 

Studies exploring predictors of outcome in HLOCs have yielded mixed findings; specifically, some work has suggested worse outcomes associated with more severe symptoms and lower body weights [27–34], AN diagnosis [20], comorbid psychopathology [34], younger age [20], higher parental expressed emotion [35], lower perceived parental control [19], and less early change in symptoms and alliance [27, 30, 32, 36]. On the other hand, several studies have found no effect of ED diagnosis [17, 28, 31, 37], symptom severity or lower body weight [31, 38], length of illness [28], or family factors [31, 32] on outcome. Finally, some work on predictors has yielded directly conflicting findings; for instance, some work has found no effect of depression or anxiety symptoms on outcome [31], other work has found that lower depression and anxiety are associated with better outcomes [34, 39], and other studies have suggested that *higher* levels of depression relate to better outcome in those with other specified EDs [20, 21, 34]. 

Despite increasing data on treatment outcomes for HLOCs in adolescents with EDs, there are some limitations of this research, which may contribute to conflicting findings. First, sample sizes for these investigations are generally small; for instance, a recent scoping review on adolescent day programs indicated that 70% of studies had sample sizes under 100, and almost a quarter of studies (24%) had sample sizes below 30 [14]. In a review of 19 studies reporting outcomes from residential treatment, only 21% of the studies had sample sizes above 300, several of which were reporting on the same sample [40]. Thus, it is likely that many existing studies have been underpowered to detect effects, particularly when exploring heterogeneity in treatment response and/or predictors of outcome. A recent simulation study focused on predicting between-patient differences in treatment for depression suggested that at least 300 individuals per arm would be required to detect marginal improvements in treatment response [41]. Secondly, many past studies on HLOCs—particularly residential treatment— have reported on combined adolescent and adult samples, or only adult samples. Considering potential developmental and clinical differences in these groups [42, 43], differences in treatment approach [44], and past data supporting differential outcomes by age of onset [43, 45], there is strong rationale to explore outcomes separately by age group when feasible, to better guide clinical decision-making and decrease risk for a more chronic course of illness. Finally, existing samples are often diagnostically homogeneous, limiting external validity of findings; for instance, the majority of existing work has focused on adolescents with restrictive EDs, most commonly AN [14, 46]. Altogether, there is a need for additional well-powered, representative samples of adolescents with heterogeneous EDs to provide a clearer picture of treatment outcome in HLOCs.

## The current study

The aims of the current study were twofold. First, we aimed to evaluate changes in self-reported ED symptoms, depression, anxiety, and objectively-measured body weight in adolescents with transdiagnostic EDs during intensive treatment. We hypothesized that, consistent with past research [14, 23, 30], adolescents would demonstrate clinical benefit (decreases in ED symptoms, depression, anxiety, binge eating episodes, and self-induced vomiting, and increases in objectively-measured body weight among those with AN) from intake to discharge. Second, we aimed to test predictors of change in outcome variables (ED symptoms; depression; anxiety; body weight; binge eating episodes; self-induced vomiting), including AN subtype, psychiatric comorbidities, ED diagnosis, age, and severity of ED symptoms at admission. Consistent with past meta-analyses [45], we expected that the binge/purge subtype of AN, psychiatric comorbidity, and older age would be associated with more severe symptoms at discharge.

## Methods

### Participants

Participants (*N* = 1,971) were youth aged 9–18 years (*M* = 14.84, *SD* = 1.64) who enrolled in an ED treatment center at one of 25 geographically distinct treatment locations across the United States, albeit all part of the same national ED company. The sample was comprised of mostly white (80.6%), cisgender females (85.3%) diagnosed with AN (60.6%). Demographic characteristics of the sample are available in Table 1.

**Table 1:** Demographic information (*N* = 1,971)

Variable	n (%)	Variable	n (%)
**Gender Identity**		**Comorbid Psychiatric Dx**	
Cisgender Female	1683 (85.3%)	Yes	1571 (79.7%)
Cisgender Male	140 (7.1%)	No	328 (16.6%)
Non-Binary	97 (4.9%)	Missing	72 (3.7%)
Transgender Female-to-Male	40 (2.0%)	**Specific Comorbidities**	
Transgender Male-to-Female	9 (0.5%)	Mood Disorder	1179 (62.1%)
Prefer Not to Answer	3 (0.3%)	Anxiety Disorder	1339 (70.5%)
**Racial Identity**		OC-Spectrum Disorders	178 (4.1%)
American Indian/Alaskan Native	5 (0.3%)	Substance Use Disorders	59 (3.1%)
Asian	75 (3.8%)	Trauma-Related Disorders	87 (4.6%)
Black or African American	28 (1.4%)	ADHD	119 (6.3%)
Hispanic or Latino	167 (8.5%)	Other	44 (2.3%)
Native Hawaiian/Pacific Islander	3 (0.2%)	**Level of Care (LOC): Admission**	
White/Caucasian	1589 (80.6%)	Inpatient	301 (15.3%)
Multiracial/Mixed Race	97 (4.9%)	Residential	858 (43.5%)
Declined to Answer/Other	1 (0.1%)	Partial Hospitalization	692 (35.1%)
Missing/Unknown	6 (0.4%)	Intensive Outpatient	120 (6.1%)
**Sex**		**Discharge Reason**	
Female	1818 (92.2%)	Administrative	85 (4.3%)
Male	153 (7.8%)	Against Medical Advice	18 (0.9%)
**Diagnosis**		Resource Constraint	29 (1.5%)
AN	1193 (60.6%)	Patient/Parent Request	375 (19.0%)
AN-R	915 (46.4%)	Routine	1315 (66.7%)
AN-BP	278 (14.1%)	COVID-19 Related	24 (1.3%)
BN	112 (5.7%)	Required Other Care	94 (4.8%)
BED	37 (1.9%)	Other	31 (1.7%)
OSFED	621 (31.5%)	**Frequency of Participation in Different LOCs**	
UFED	8 (0.4%)	Inpatient	352 (17.9%)
		Residential	1185 (60.1%)
		Partial Hospitalization	1428 (72.5%)
		Intensive Outpatient	796 (40.4%)

Note. AN = anorexia nervosa (R or BP for restrictive or binge/purge subtype, respectively); BN = bulimia nervosa; BED = binge eating disorder; OSFED = other specified feeding and eating disorder; UFED = unspecified feeding and eating disorder; OC-spectrum = obsessive-compulsive-spectrum; ADHD = attention-deficit/hyperactivity disorder

### Measurements

*Diagnoses.* ED diagnoses and psychiatric comorbidities were ascertained through semi-structured interviews informed by the Diagnostic and Statistical Manual for Mental Disorders, 5th Edition [47] criteria; interviews were conducted by treatment center staff with master’s degrees. For the purposes of analysis, the research team pulled these data from each participant’s medical chart, and psychiatric comorbidity was coded as “present” or “absent.”

*Eating Disorder Examination—Questionnaire (EDE-Q)* [48]. Participants completed the 31-item EDE-Q, which is widely used as a self-report measurement of ED behaviors and symptoms. Within the current analyses, we used the EDE-Q Global score as an indicator of overall ED symptoms. Internal consistency for the measure in our sample was excellent across timepoints (Cronbach’s α = 0.96-0.97).

*Patient Health Questionnaire (PHQ-9)* [49]. We used the PHQ-9 to measure depressive symptoms. The PHQ-9 contains nine items gauging depressive symptoms over the past 2 weeks, rated on a Likert-type scale ranging from 0 (“Not at all”) to 3 (“Nearly Every Day”). Reliability in our sample over timepoints was excellent (Cronbach’s α = 0.86-0.89).

*Generalized Anxiety Disorder-7 (GAD-7)* [50]. The GAD-7, a 7-item self-report scale gauging broad anxiety-based symptoms, was used as a measurement of anxiety. Items are rated using a Likert-type scale ranging from 0 (“Not at all”) to 3 (“Nearly Every Day”). Scores range from 0 to 21, with higher scores indicating greater anxiety. Reliability in our sample across timepoints was excellent (Cronbach’s α = 0.90-0.92).

*Percentage of estimated body weight.* To provide a measure of estimated body weight, we divided the observed weight of participants (weighed during their time in treatment) by their expected body weight based on gender, age, and historical growth curve data, as is recommended [51]. Body weight data were available at intake and discharge.

### Treatment description

Treatments were provided at the IP, RES, PHP, and IOP levels of care. Admitted participants were assigned to a given level of care based on clinical assessment and medical acuity; most patients in this sample were admitted at the RES (43.5%; *n* = 858) or PHP (35.1%; *n =* 692) levels. At all levels of care, participants received individual therapy two times a week (consistent with the overall treatment center approach), weekly family therapy, regular visits with a psychiatrist (daily to weekly depending on the level of care), regular visits with a dietitian (at least weekly depending on level of care), as needed visits with a medical doctor, and group therapy sessions based on evidence-based modalities, including cognitive behavioral therapy, dialectical behavior therapy, emotion-focused family therapy, and acceptance and commitment therapy. Consistent with data supporting family-based treatment as the first-line treatment in youth [52], parents and caregivers were engaged in programming within a “family-empowered” model that involved regular communication throughout all levels of care, regular meetings with providers, emphasis on caregiver-led decisions for their child’s nutrition during treatment, promotion of caregiver alignment, and caregiver presence at meals or snacks in treatment [53]. Family-based therapeutic contact was additionally influenced by Emotion-Focused Family Therapy [54]. Participants in IP, RES, and PHP programming received three supervised meals and two to three supervised snacks daily. Participants in IOP had one supervised meal per day.

### Procedures

The current study was approved by Salus Institutional Review Board. All participants who admitted during the timeframe of data collection (October 2020-January 2023) were considered eligible for the study. Thus, all new patients and their parents/legal guardians were approached at admission to provide informed consent and assent for participation in the current study. Following provision of informed consent/assent, participants completed self-report questionnaires at intake to treatment, first step-down to a lower level of care, and discharge from treatment (i.e., within 7 days of discharge). Of note, there was significant missingness in the dataset; 23.3% (*n* = 302) of those who were eligible to complete a stepdown assessment (i.e., 1,292 participants stepped down to at least one other level of care) were missing, and 55.6% of participants (*n* = 1,095) were missing discharge self-report measurements. We only analyzed data for those who completed admission assessments. For details on patterns in missingness, please see the Supplement; comparisons across study variables indicated more consistent patterns in missingness at discharge, with some differences according to discharge type, gender, race, ED diagnosis, psychiatric comorbidity, and %EBW at admission. Overall, those with lower %EBW at admission, cisgender females, those with AN-R, psychiatric comorbidity, and routine discharges were more likely to have complete data. Importantly, tests indicated that those with missing stepdown or discharge data did not endorse more severe baseline symptoms (as measured by the EDE-Q, binge eating/vomiting episodes, and %EBW at entry to treatment). However, results do suggest patterns in missingness related to demographic variables (race/ethnicity; gender) or care entry variables (LOC at admission; reason for discharge) that are important to keep in mind when interpreting findings.

### Statistical analysis plan

For participants with multiple treatment admissions during the data collection period (*n* = 95, 4.3%), we only used data from their first treatment stay, consistent with other studies exploring treatment outcomes at HLOCs [23, 55]. Due to variability in the admitting level of care, we elected to analyze data from (a) individuals who admitted to IP or RES treatment and (b) those who admitted to PHP or IOP separately for initial models. Given similarity in results across these initial models, we conducted predictor analyses within the full sample to maximize power.

*Aim 1.* To explore our first aim regarding changes in ED symptoms, depression, anxiety, and %EBW throughout treatment, we used multi-level models, implemented in R using the *lme4* package [56]. Across models, we controlled for age at admission and length of stay. We used the *r2glmm* package in R to estimate effect sizes, expressed as a partial *R* [57]. To explore changes in binge eating symptoms among those with AN-BP, BN, and BED and self-induced vomiting in those with AN-BP and BN, we ran negative binomial mixed models using the *glmmTMB* package in R [58], controlling for length of stay and age. Of note, two participants self-reported binge eating and vomiting episode frequency that was unlikely to be valid (i.e., episodes > 500) and were removed within subsequent analyses. We used full-information maximum likelihood estimation to handle missing data, consistent with research suggesting that this method yields non-biased estimates when data is missing-at-random [59, 60]. Given variability in timing of assessment points across individuals, time was coded as days since admission.

To calculate the percentage of participants reporting reliable change in symptoms, we used the reliable change index (RCI) outlined by Jacobson and Truax [61], and thus use the term “reliable change” to mean change on a self-report measurement that is larger than would be expected for a difference caused by error alone. Using this approach and based on the admission standard deviation and reliability of each scale, participants who completed measurements and reported change greater than 0.88 points change in the EDE-Q global, 6.51 points on the PHQ-9, and 5.03 on the GAD-7 were determined to evidence “reliable” change. We defined clinically-significant change using Jacobson and Truax’s clinical significance criterion “c,” and thus use the term to indicate when a participant moved from endorsing a score outside of the "normal range" to endorsing a score within the "normal range." To operationalize the "normal range," we used adolescent community norms for each measurement [61–64]. Clinically-significant change was defined as moving from a score above 2.71 points on the EDE-Q, 9.48 points on the PHQ-9, and 7.97 points on the GAD-7 to a score below these thresholds. Use of these metrics allowed us to categorize individuals into five groups: (1) normative (scores in the normative range at both admission and discharge), (2) deteriorated (reliable worsening of symptoms), (3) unchanged (scores above norm at admission and statistically non-significant individual changes at discharge), (4) improved (making reliable change but not endorsing clinically significant change), and (5) clinically significant change (endorsing both reliable change and clinically significant change).

*Aim 2.* To test predictors of treatment-related change, we ran multi-level models exploring interactions between time and predictor variables (AN-BP subtype, age, admission EDE-Q global scores, ED diagnosis, and comorbid psychiatric diagnoses). ED diagnoses (BN; BED; other specified eating disorder [OSFED]/unspecified eating disorder [UFED]) were coded into categorical variables using simple coding, with AN as the reference group. Given the number of models tested across aims, we used a Bonferroni correction for determining the statistical significance threshold (0.05/17 = 0.003). In response to a reviewer comment regarding site effects, we conducted post-hoc analyses exploring the effect of geographical region of treatment site, coded as a dummy variable based on U.S. Census regions (e.g., Midwest; Northeast; South; West, with South as the reference group), and because of unequal representation of levels of care across regions, also controlled for levels of care within these analyses.

## Results

Means, standard deviations, and ranges for all study variables are available in Table 2. At admission, participants reported ED symptoms at levels that were around 1.5 standard deviations above adolescent community norms [63, 65], consistent with other reports of EDE-Q global scores in youth with EDs presenting to HLOCs [14]. Participants endorsed a mean level of depressive symptoms in the moderately severe range and anxiety symptoms in the moderate range. Total mean length of stay was 79.97 days (*SD* = 49.56 days), and mean length of stay at each level of care ranged from 25.93 days for IP treatment (*SD* = 20.76) to 45.49 days in IOP (*SD* = 22.16).

**Table 2:** Descriptive statistics

Variable	M (SD)	Range
Age at Admission	14.84 (1.64)	9–18
Admission % of EBW (Full Sample)	99.10 (24.35)	57.58-286.17
Discharge % of EBW (Full Sample)	111.80 (21.77)	65.88-288.19
Admission % of EBW (AN Only)	87.46 (10.48)	57.58–143.60
Discharge % of EBW (AN Only)	103.88 (11.91)	65.88-167.94
Length of Stay (Total)	79.97 (49.56)	1-386
Length of Stay (Inpatient)	25.93 (20.76)	0-149
Length of Stay (Residential)	42.72 (23.51)	0-170
Length of Stay (PHP)	38.75 (20.00)	0-160
Length of Stay (IOP)	45.49 (22.16)	0-136
EDE-Q Global Admission	3.62 (1.59)	0.00-5.95
EDE-Q Global Stepdown	2.33 (1.42)	0.00–6.00
EDE-Q Global Discharge	2.27 (1.52)	0.00–6.00
PHQ-9 Admission	14.87 (7.08)	0–27
PHQ-9 Stepdown	10.40 (6.48)	0–27
PHQ-9 Discharge	10.33 (6.68)	0–27
GAD-7 Admission	12.93 (5.74)	0–21
GAD-7 Stepdown	10.82 (5.90)	0–21
GAD-7 Discharge	10.61 (6.00)	0–21

Note. EBW = estimated body weight; PHP = partial hospitalization program; IOP = intensive outpatient; EDE-Q = Eating Disorders Examination– Questionnaire; PHQ-9 = Patient Health Questionnaire; GAD-7 = Generalized Anxiety Disorder

### Changes in EDE-Q global over treatment (Table 3)

**Table 3:** Fixed effects from multi-level models exploring changes in symptoms over time

Inpatient and Residential (*n* = 1,158)																	
	EDE-Q Model						PHQ-9 Model						GAD Model				
Predictor	Est.	se	*p*	*R*_*p*_^*2*^	*95%CI*		Est.	se	*p*	*R*_*p*_^*2*^	*95%CI*		Est.	se	*p*	*R*_*p*_^*2*^	*95%CI*
Intercept	**3.34**	**0.05**	**< 0.001**	**0.10**	**0.08**,** 0.12**		**13.98**	**0.20**	**< 0.001**	**0.06**	**0.04**,** 0.08**		**12.59**	**0.20**	**< 0.001**	**0.02**	**0.01**,** 0.04**
Time	**-0.01**	**0.00**	**< 0.001**	**0.09**	**0.07**,** 0.11**		**-0.04**	**0.00**	**< 0.001**	**0.05**	**0.04**,** 0.07**		**-0.02**	**0.00**	**< 0.001**	**0.02**	**0.01**,** 0.03**
Age	0.03	0.03	0.186	0.00	0.00, 0.01		0.32	0.11	0.004	0.01	0.00, 0.01		-0.01	0.11	0.895	0.00	0.00, 0.00
LOS	**0.01**	**0.00**	**< 0.001**	**0.03**	**0.02**,** 0.04**		**0.02**	**0.00**	**< 0.001**	**0.01**	**0.01**,** 0.02**		**0.01**	**0.00**	**< 0.001**	**0.02**	**0.01**,** 0.03**

Partial Hospitalization/Intensive Outpatient (*n* = 812)																	
	EDE-Q Model						PHQ-9 Model						GAD Model				
Predictor	Est.	se	*p*	*R*_*p*_^*2*^	*95%CI*		Est.	se	*p*	*R*_*p*_^*2*^	*95%CI*		Est.	se	*p*	*R*_*p*_^*2*^	*95%CI*
Intercept	**3.49**	**0.05**	**< 0.001**	**0.15**	**0.12**,** 0.18**		**14.31**	**0.24**	**< 0.001**	**0.10**	**0.08**,** 0.14**		**12.59**	**0.25**	**< 0.001**	**0.06**	**0.03**,** 0.09**
Time	**-0.02**	**0.00**	**< 0.001**	**0.14**	**0.11**,** 0.17**		**-0.07**	**0.00**	**< 0.001**	**0.09**	**0.06**,** 0.12**		**-0.04**	**0.00**	**< 0.001**	**0.04**	**0.02**,** 0.07**
Age	0.08	0.03	0.005	0.01	0.00, 0.02		**0.06**	**0.13**	**< 0.001**	**0.02**	**0.01**,** 0.04**		**0.44**	**0.13**	**0.001**	**0.02**	**0.00**,** 0.04**
LOS	**0.01**	**0.00**	**< 0.001**	**0.03**	**0.01**,** 0.05**		0.01	0.01	0.005	0.01	0.00, 0.02		0.01	0.01	0.050	0.01	0.00, 0.02

Note. LOS = Length of stay; *R*_*p*_^*2*^*=* partial R [2] effect size calculated using r2glmm package. Effects with a *p*-value < 0.003 are bolded

*IP/RES Subsample.* The model exploring changes in EDE-Q global scores over time indicated a significant effect of time and length of stay, such that EDE-Q global scores significantly decreased over time, and length of stay was positively associated with greater symptom severity across timepoints. The percentages of the reporting IP/RES adolescent sample who experienced reliable and clinically significant change in ED symptoms, depression, and anxiety are available in Table 4. Over 50% of adolescents reported reliable improvements in symptoms from intake to stepdown and intake to discharge, with over 30% of the reporting sample noting improvements that were clinically significant. Around 20% of the sample reported no change in symptoms, around 25% reported symptoms in the normative range at both intake and discharge, and under 5% reported worsening of symptoms.

**Table 4:** Percentage of reporting samples reporting reliable and clinical change note. Percentages represent the number of participants within each category within the reporting sample

Inpatient/Residential Sample						
		CSC %^a^	Reliable %^b^	Unchanged %^c^	Deteriorated %^d^	Normative %^e^
EDEQ	Admit to Stepdown	32.0%	21.0%	22.0%	3.0%	22.0%
	Admit to Discharge	34.3%	16.4%	20.7%	4.0%	24.6%
PHQ-9	Admit to Stepdown	24.3%	11.4%	44.8%	3.1%	16.3%
	Admit to Discharge	22.0%	10.2%	38.7%	5.7%	23.3%
GAD-7	Admit to Stepdown	12.2%	10.9%	57.0%	7.6%	12.2%
	Admit to Discharge	12.8%	10.0%	54.8%	6.7%	15.8%

PHP/IOP Sample						
		CSC %^a^	Reliable %^b^	Unchanged %^c^	Deteriorated %^d^	Normative %^e^
EDEQ	Admit to Stepdown	39.8%	16.3%	22.3%	0.6%	21.1%
	Admit to Discharge	33.3%	14.9%	24.9%	1.6%	25.3%
PHQ-9	Admit to Stepdown	27.0%	11.9%	38.0%	2.1%	21.1%
	Admit to Discharge	21.1%	14.2%	40.9%	1.2%	22.7%
GAD-7	Admit to Stepdown	20.1%	10.6%	50.3%	3.7%	15.3%
	Admit to Discharge	13.6%	12.3%	55.2%	2.6%	16.2%

^a^Among those in the clinical range, participants’ scores met the RCI criteria and crossed the clinical cut-off score into the normative range

^b^Among those in the clinical range, participants’ scores met the RCI criteria but did not cross the cut-off score

^c^Among those in the clinical range, participants’ scores did not meet the RCI criteria

^d^Among those in the clinical range, participants’ scores met the RCI criteria in the opposite direction (worsened)

^e^Participants’ scores fell within the normal range of functioning at both timepoints

*PHP/IOP subsample*. Models in the PHP and IOP subsample demonstrated consistent results with IP/RES; specifically, there was a significant main fixed effect of time, suggesting decreasing EDE-Q global scores over time, alongside a significant effect of length of stay, with longer length of stay associated with more severe ED symptoms.

Metrics for reliable and clinical change in symptoms in the PHP/IOP subsample are available in Table 4. In a similar manner as those admitting to IP and IP treatment, around 50% of the sample reported reliable improvements in EDE-Q global scores at stepdown and/or discharge, with around 35% of the reporting sample noting improvements that were clinically significant. Similar to patterns observed in the IP and RES sample, around 25% of the sample reported symptoms in the normative range at both timepoints, just over 20% reported no change in symptoms, and less than 2% reported worsening of symptoms.

### Changes in anxiety and depression over treatment (Table 3)

*IP/RES Subsample.* The model exploring changes in PHQ-9 scores over time in those admitting to IP and RES programs indicated a significant effect of time and length of stay, such that depressive symptoms generally decreased over time and were higher among those with longer lengths of stay. In a parallel manner, the GAD-7 models suggested significant decreases in anxiety over time and higher anxiety among those with longer length of stay.

Calculation of the RCI and clinical significance metrics for PHQ and GAD scores suggested consistent patterns (see Table 4). Regarding depressive symptoms, around 35% of the sample reported reliable improvements in depressive symptoms at stepdown and/or discharge, with over 20% of participants reporting clinically significant change. Around 20% of the sample reported normative depressive scores at intake and discharge, around 40% remained unchanged throughout treatment, and around 5% of the sample reported worsening of symptoms. GAD-7 scores indicated under 30% of the sample reporting reliable improvement in symptoms at stepdown or discharge, and around 15% reporting clinically significant changes. Around 20% reported normative levels of anxiety at both timepoints, 55% of the sample reported no change in their anxiety over treatment, and around 7% reported deterioration in symptoms.

*PHP/IOP Subsample.* Multi-level models exploring changes in PHQ-9 and GAD-7 scores in the PHP/IOP subsample suggested significant effects of time, such that symptoms decreased over time, and age, with older age at admission relating to higher mean symptoms across timepoints.

Reliable and clinical change metrics for the PHP/IOP sample (Table 4) suggested that just over 35% of the sample reported reliable improvements in depression at stepdown or discharge, with around 10% of the reporting sample noting changes that were clinically significant. Around 20% reported normative symptoms at both timepoints, 40% remained unchanged in their depressive symptoms across timepoints, and around 2% reported deterioration. GAD-7 scores evidenced a similar pattern, wherein around 25% of the sample reported reliable improvements in anxiety across timepoints, with about 15% of the sample reporting changes that were clinically significant. Around 15% of the sample reported normative anxiety across timepoints, 50% of the sample reported no change in symptoms, and around 3% of the sample reported deterioration of symptoms.

### Changes in body weight over time (AN only; Table 5)

**Table 5:** Fixed effects from multi-level models exploring changes in %EBW over time

Inpatient/Residential (*n* = 791)					
Predictor	Est.	*SE*	*p*	*R*_*p*_^*2*^	*95%CI*
Intercept	**86.56**	**0.36**	**< 0.001**	**0.50**	**0.46**,** 0.53**
Time	**0.23**	**0.01**	**< 0.001**	**0.40**	**0.45**,** 0.52**
Age	**-1.38**	**0.21**	**< 0.001**	**0.04**	**0.03**,** 0.07**
LOS	**-0.06**	**0.01**	**< 0.001**	**0.06**	**0.04**,** 0.10**

Partial Hospital/Intensive Outpatient (*n* = 394)					
Predictor	Est.	*SE*	*p*	*R*_*p*_^*2*^	*95%CI*
Intercept	**91.16**	**0.52**	**< 0.001**	**0.25**	**0.19**,** 0.31**
Time	**0.16**	**0.01**	**< 0.001**	**0.20**	**0.15**,** 0.26**
Age	**-1.59**	**0.31**	**< 0.001**	**0.04**	**0.03**,** 0.11**
LOS	**-0.04**	**0.01**	**< 0.001**	**0.08**	**0.01**,** 0.07**

Note. %EBW = percent of expected body weight; LOS = length of stay. *R*_*p*_^*2*^*=* partial R^2^ effect size calculated using r2glmm package. Effects with a *p*-value < 0.003 are bolded

*IP/RES Subsample.* Results from a mixed model exploring changes in percent of expected body weight (%EBW) suggested a significant fixed effect of time, such that participants with AN demonstrated increases in %EBW from intake to discharge. Participants evidenced, on average, an increase of 16.43% EBW from admission to discharge (*SD* = 10.87). Age and length of stay also demonstrated significant fixed effects, such that younger age at admission and shorter length of stay were associated with higher mean body weight across timepoints.

*PHP/IOP Subsample.* Within the PHP/IOP subsample, the model demonstrated a parallel pattern of results as the IP/RES sample, such that there were significant mean increases in %EBW over time, with younger admission age and shorter length of stay significantly predicting higher %EBW across timepoints.

### Binge eating & vomiting models (Table 6)

**Table 6:** Fixed effects from multi-level models exploring changes in binge eating and self-induced vomiting symptoms over time

Inpatient/Residential								
Predictor		Binge Eating Model (*n* = 255)				Vomiting Model (*n* = 245)		
		Est.	*SE*	*p*		Est.	*SE*	*p*
Intercept		**1.44**	**0.18**	**< 0.001**		**2.76**	**0.36**	**< 0.001**
Time		**-0.04**	**0.01**	**< 0.001**		**-0.08**	**0.01**	**< 0.001**
Age		-0.16	0.10	0.101		0.00	0.20	0.992
LOS		0.00	0.01	0.128		0.01	0.01	0.123

Partial Hospital/Intensive Outpatient								
Predictor		Binge Eating Model (*n* = 172)				Vomiting Model (*n* = 145)		
		Est.	*SE*	*p*		Est.	*SE*	*p*
Intercept		**1.52**	**0.19**	**< 0.001**		**2.07**	**0.14**	**< 0.001**
Time		**-0.04**	**0.01**	**< 0.001**		**-0.02**	**0.00**	**< 0.001**
Age		0.03	0.09	0.343		0.12	0.09	0.189
LOS		0.00	0.00	0.857		0.00	0.00	0.480

Note. LOS = Length of stay. Effects with a *p*-value < 0.003 are bolded

*IP/RES subsample.* Models exploring changes in binge eating episodes and self-induced vomiting among those admitting to IP/RES suggested significant effect of time, such that binge eating and self-induced vomiting episodes both decreased over time. The effects of age and length of stay were not significant.

*PHP/IOP subsample*. In those admitting to PHP/IOP, there was a significant effect of time for both binge eating and self-induced vomiting, such that binge episodes and self-induced vomiting episodes decreased over time, with no significant effects of age or length of stay.

### Predictors of outcome (Table 7)

*EDE-Q global model.* Results from the model exploring changes in EDE-Q global scores over treatment indicated significant fixed effects of time, length of stay, and admission EDE-Q global scores. Regarding interaction effects, there were significant interactions between time and psychiatric comorbidity, AN binge/purge subtype, BN and OSFED diagnoses, and admission EDE-Q scores. Graphical representation of predicted values for these interactions suggested that those with no psychiatric comorbidity and higher EDE-Q global scores at admission demonstrated greater reductions in ED symptoms over treatment. Diagnosis-specific interactions suggested that having a diagnosis of AN-BP was associated with greater reductions in ED symptoms; further, those with BN and OSFED endorsed greater change in EDE-Q global scores over time compared to those with AN.

**Table 7:** Estimates of fixed effects from predictor models (full sample)

Predictor	EDE-Q Model					PHQ-9 Model					GAD Model				
	Est	*SE*	*p*	*R*_*p*_^*2*^	*95%CI*	Est	*SE*	*p*	*R*_*p*_^*2*^	*95%CI*	Est	*SE*	*p*	*R*_*p*_^*2*^	*95%CI*
Intercept	**3.49**	**0.06**	** < .001**	**.64**	**.62, .66**	**12.72**	**0.35**	** < .001**	**.34**	**.31, .36**	**11.82**	**0.37**	** < .001**	**.25**	**.22, .28**
Time	**-0.02**	**0.00**	** < .001**	**.03**	**.02, .05**	**-0.05**	**0.01**	** < .001**	**.01**	**.01, .02**	**-0.02**	**0.01**	** < .001**	**.01**	**.00, .01**
EDEQ Admission	**0.90**	**0.01**	** < .001**	**.56**	**.54, .57**	**2.64**	**0.08**	** < .001**	**.25**	**.22, .27**	**1.82**	**.09**	** < .001**	**.17**	**.14, .19**
Age	0.01	0.01	.601	.00	.00, .00	**0.30**	**0.01**	** < .001**	**.00**	**.00, .01**	0.09	0.09	.296	.00	.00, .01
LOS	**0.00**	**0.00**	** < .001**	**.02**	**.01, .03**	0.01	0.00	.003	.00	.00, .01	0.01	0.00	.003	.01	.00, .01
AN-BP^1^	-0.03	0.06	.557	.00	.00, .00	0.35	0.40	.379	.00	.00, .00	-0.25	0.41	.549	.00	.00, .00
Comorbid	-0.17	0.05	.003	.00	.00, .01	**1.15**	**0.35**	**.001**	**.00**	**.00, .01**	1.04	0.37	.005	.00	.00, .01
BN^2^	0.12	0.10	.233	.00	.00, .00	0.99	0.60	.101	.00	.00, .00	-0.22	0.61	.715	.00	.00, .00
BED^2^	-0.05	0.16	.739	.00	.00, .00	0.82	0.99	.411	.00	.00, .00	0.17	1.15	.884	.00	.00, .00
OSFED/UF^2^	0.06	0.05	.234	.00	.00, .00	0.78	0.30	.011	.00	.00, .01	-0.01	0.33	.954	.01	.00, .01
Time x Age	-0.00	0.00	.525	.00	.00, .00	-0.00	0.00	.149	.00	.00, .00	-0.00	0.00	.203	.00	.00, .00
Time x AN-BP^1^	**-0.01**	**0.00**	** < .001**	**.00**	**.00, .01**	-0.01	0.01	.058	.00	.00, .00	-0.01	0.01	.105	.00	.00, .00
Time x Comorbid	**0.01**	**0.00**	** < .001**	**.00**	**.00, .01**	0.01	0.01	.105	.00	.00, .00	0.01	0.01	.150	.00	.00, .00
Time x BN^2^	**-0.02**	**0.00**	** < .001**	**.01**	**.01, .02**	**-0.09**	**0.02**	** < .001**	**.01**	**.00, .01**	**-0.05**	**0.01**	** < .001**	**.00**	**.00, .01**
Time x BED^2^	-0.01	0.01	.019	.00	.00, .01	-0.05	0.03	.112	.00	.00, .00	-0.04	0.03	.209	.00	.00, .00
Time x OSFED^2^	**-0.01**	**0.00**	** < .001**	**.01**	**.01, .02**	**-0.02**	**0.02**	** < .001**	**.00**	**.00, .01**	-0.01	0.01	.057	.00	.00, .01
Time x EDEQ Admission	**-0.01**	**0.00**	** < .001**	**.07**	**.06, .09**	**-0.02**	**0.00**	** < .001**	**.02**	**.02, .03**	**-0.00**	**0.00**	** < .001**	**.00**	**.00, .01**

*Note.* LOS = length of stay; AN-BP = binge/purge subtype of AN; BN = bulimia nervosa; BED = binge eating disorder; OSFED/UF = other specified ED/unspecified ED, EDE-Q Admission = EDE-Q Global score at baseline. *R*_*p*_^*2*^ = partial R^2^ effect size calculated using r2glmm package. Effects with a *p*-value < .003 are bolded

^1^This effect refers to the impact of having an AN-BP diagnosis on the outcome, compared to having all other diagnoses

^2^Diagnosis-specific effects indicate the impact of having the listed diagnosis on the outcome, compared to those with AN (reference group)

*PHQ-9 model.* The PHQ-9 model suggested significant fixed effects of time, age, psychiatric comorbidity, and admission EDE-Q scores, such that those with older age, psychiatric comorbidity, and higher EDE-Q admission scores had higher mean PHQ-9 scores across timepoints. There were also significant interaction terms, such that those with higher EDE-Q admission scores demonstrated greater decreases in depression scores over time; further, significant interactions relative to diagnosis indicated that those with BN and OSFED demonstrated greater change in depressive symptoms, compared to those with a diagnosis of AN.

*GAD-7 model.* The GAD-7 model suggested significant fixed effects of time and admission EDE-Q scores, such that those with higher EDE-Q global scores at admission demonstrated higher GAD-7 scores across timepoints. Interaction terms suggested that those with BN and higher EDE-Q global scores demonstrated steeper reductions in GAD-7 scores over time.

*%EBW model (*Table 8*).* The EBW model indicated significant fixed effects of time, age, length of stay, binge/purge subtype, and admission EDE-Q global scores, such that those with older age, longer length of stay, binge/purge subtype, and lower EDE-Q global scores had lower %EBW across time points. There were significant interactions between time and EDE-Q global scores at admission, such that those with higher EDE-Q global scores demonstrated greater *increases* in body weight over time.

**Table 8:** Estimates of fixed effects from predictor models for %EBW

Predictor	Est.	SE	*p*
Intercept	**87.33**	**0.73**	**< 0.001**
Time	**0.19**	**0.01**	**< 0.001**
EDEQ Admission	**0.81**	**0.19**	**< 0.001**
Age	**-1.57**	**0.19**	**< 0.001**
LOS	**-0.06**	**0.01**	**< 0.001**
AN-BP^1^	**4.56**	**0.71**	**< 0.001**
Comorbid	-0.30	0.78	0.701
Time x Age	-0.01	0.00	0.024
Time x AN-BP^1^	-0.01	0.01	0.337
Time x Comorbid	0.03	0.01	0.021
Time x EDEQ Admission	**-0.01**	**0.00**	**< 0.001**

Note. %EBW = percent of expected body weight; LOS = length of stay; AN-BP = anorexia nervosa, binge/purge subtype; EDEQ Admission = Eating Disorder Examination-Questionnaire Global score at baseline. Effects with a *p*-value < 0.003 are bolded

^1^This effect refers to the impact of having an AN-BP diagnosis on body weight, compared to AN-R

*Binge eating episodes (*Table 9*).* Models exploring predictors of binge episode frequency suggested significant effects of BN, BED, and EDE-Q global scores at admission, such that those with BN and BED reported more frequent binge episodes compared to those with AN-BP, and those with higher EDE-Q global scores at admission also reported more frequent episodes across timepoints. No effects met our adjusted *p*-value criteria, suggesting no reliable predictors of change in binge episodes over time.

**Table 9:** Estimates of fixed effects from predictor models for binge eating and self-induced vomiting

Predictor		Binge Eating Model (*n* = 405)				Vomiting Model (*n* = 414)		
		Est.	*SE*	*p*		Est.	*SE*	*p*
Intercept		0.87	032	0.006		**2.07**	**0.27**	**< 0.001**
Time		**-0.05**	**0.01**	**< 0.001**		**-0.05**	**0.01**	**< 0.001**
EDEQ Admission		**0.28**	**0.08**	**0.001**		**0.22**	**0.07**	**0.001**
Age		0.07	0.08	0.406		0.05	0.07	0.437
LOS		0.00	0.00	0.118		0.01	0.00	0.012
AN-BP^1^		--	--	--		-0.22	0.22	0.313
Comorbid		-0.00	0.31	0.989		0.05	0.28	0.867
BN^2^		**1.01**	**0.38**	**< 0.001**		--	--	--
BED^2^		**1.99**	**0.42**	**< 0.001**		--	--	--
Time x Age		0.00	0.00	0.210		0.00	0.00	0.085
Time x AN-BP^1^		--	--	--		0.02	0.01	0.010
Time x Comorbid		0.01	0.01	0.555		0.02	0.01	0.036
Time x BN^1^		-0.00	0.01	0.678		--	--	--
Time x BED^1^		-0.02	0.02	0.301		--	--	--
Time x EDEQ Admission		-0.00	0.00	0.367		**0.02**	**0.00**	**< 0.001**

Note. LOS = length of stay; AN-BP = anorexia nervosa, binge/purge subtype; BN = bulimia nervosa; BED = binge eating disorder; EDEQ Admission = Eating Disorder Examination-Questionnaire Global score at baseline. Effects with a p-value < 0.003 are bolded

^1^This effect refers to the impact of having an AN-BP diagnosis on the outcome, compared to having BN

^2^ Diagnosis-specific effects indicate the impact of having the listed diagnosis on the outcome, compared to those with AN-BP (reference group)

*Vomiting episodes (*Table 9*).* Self-induced vomiting models indicated a significant main effect of time and EDE-Q global scores, suggesting that those with higher admission EDE-Q global scores reported more frequent vomiting episodes across timepoints. The interaction between EDE-Q scores at admission and time was significant, suggesting that those with higher EDE-Q scores demonstrated less change in vomiting over time. No other effects reached our statistical significance threshold.

Post-hoc analyses (see Supplement). Full results from post-hoc analyses exploring the effect of level of care and geographical region on change in symptoms can be found in the Supplement. Overall, the pattern of observed results remained the same, with some minor changes. Specifically, within the PHQ predictor model, the OSFED predictor was no longer significant (*p* =.007). Within the GAD model, baseline EDE-Q scores were no longer significant predictors of symptom change (*p* =.004). There were no changes in the binge eating and %EBW models. Finally, within vomiting models, interactions between the AN-BP subtype and time and comorbidity and time were significant, suggesting those with BN and no comorbidity demonstrated more steep declines in vomiting over time. Across these models, interactions between time and the Western region emerged, suggesting that those in treatment centers within the US Western region demonstrated slightly less change in symptoms compared to those from the South.

## Discussion

Although HLOCs are frequently used in the management of ED symptoms in adolescents within the United States [14, 40], data on the effectiveness of these programs remain surprisingly limited in scope. In the current manuscript, we report outcomes and predictors of outcome for a large sample of treatment-seeking adolescents with EDs participating in HLOCs across the United States. Overall, our findings were consistent with past studies in this area, suggesting broad clinical benefit associated with participating in intensive programming, particularly in ED symptoms; however, results also suggested variability in treatment effects, highlighting important domains for future study.

Regarding our first aim, models exploring changes in global ED symptoms, anxiety, binge eating, self-induced vomiting, and depression suggested significant reductions in these symptoms from admission to discharge after controlling for length of stay and participant age. Models exploring changes in body weight among those with AN also suggested benefit over time, with significant increases in percent of expected body weight. Our findings are consistent with past studies in adolescent-focused intensive treatments [14, 23, 30, 66] and mixed samples of adults and adolescents [26, 40] and extend this prior research in two ways. First, our sample represents the largest sample of adolescents in HLOC to date, increasing confidence in past effects. Second, individuals in the current study, while predominantly composed of those with AN, included higher numbers of those with other ED diagnoses than have been included in past work.

Analyses exploring the proportion of individuals achieving reliable change in symptoms provide additional context to our findings. Across levels of care, use of the RCI suggested that around half of the sample reported reliable improvements in ED symptoms over the course of treatment, with the remainder of the sample reporting no reliable change in symptoms or consistently reporting scores in the normative range. Very few individuals among the reporting sample (< 5%, *range* = 22–40 participants per outcome) reported deterioration of symptoms. Our rates of reliable and clinically-significant change are consistent with past work [20, 34, 67–69], suggesting that clinical deterioration in HLOCs is rare and that there are significant benefits for many. However, they also highlight a consistent opportunity to improve effectiveness in HLOCs and several important directions for future research, including the need to more comprehensively characterize those who did not evidence clinical benefit and designing adjunctive interventions for these groups. For instance, given aim 2 findings indicated that those with psychiatric comorbidity experienced less change in ED symptoms over treatment, future work might consider exploring the benefit of incorporating treatment content focused on co-occurring symptoms, such as published protocols for targeting comorbidities in ED samples [70–74]. 

As another important point, while our findings support the effectiveness of HLOC for adolescents with EDs, our naturalistic design did not permit direct comparisons of outcomes and costs associated with standard outpatient treatment for adolescent EDs, given lack of a matched comparison group. Both questions (i.e., direct comparison of outcomes and cost of HLOC versus outpatient treatment) have been somewhat neglected by past work (for exceptions, see [75–79]), and represent critical questions to be answered by future research. Relatedly, while published guidelines do exist regarding decision-making in referring a client for an ED-focused HLOC or stepping up and down in care intensity [80–83], there are few empirical data supporting comprehensive clinical decision-making frameworks. We recommend that future work (a) more comprehensively characterize how these decisions are currently made across different care settings, providers, and countries, and (b) empirically evaluate different frameworks for effectively making these decisions, ideally using large-scale, representative samples and cohort designs.

It is notable that around 25–30% of our sample reported EDE-Q scores in the normative range at both intake and discharge, which may also account for a lack of robustly positive findings regarding reliable and clinical change. Notably, these rates are similar to others reported in the literature [20, 69]; past research has suggested that around 40% of treatment-seeking adolescents with EDs either deny or minimize symptoms [84, 85]. Past work has suggested that low levels of endorsement may reflect several phenomena, such as individuals with limited insight into their illness, ambivalence about the need for treatment, ED presentations lower in shape and weight concerns (including non-fat-phobic AN), or those motivated to minimize symptoms for other reasons, such as attempts to avoid hospitalization or treatment for their ED, shame, or rationalization of their behavior as healthy [84, 86–89]. 

Analysis of reliable change in PHQ-9 and GAD-7 scores suggested that between a quarter to one-third of the sample reported reliable and/or clinically meaningful improvement in anxiety and depression. Past reports of reliable clinical change in depressive symptoms has yielded rates ranging from 25–40%.^20,67^ Notably, we set a somewhat stringent threshold for determining reliable and clinical change; however, when recalculating reliable change in the PHQ-9 and GAD-7 using other thresholds implemented in past literature (e.g., ≥ 5.2 point change in PHQ-9; ≥ 3.53 point change in GAD-7^90^), rates of reliable symptom improvement reached around 40%, which fall within the ranges reported in prior literature. Lower rates of change in anxiety and depression in our sample compared to ED symptoms may simply be a function of the programs' primary focus on disordered eating. It may also reflect the stage of recovery associated with enrolling in intensive treatment; more specifically, most individuals enrolling in HLOCs are discharged to outpatient or other intensive treatments for ongoing support. Therefore, it could be the case that improvements in these symptoms occur later in the course of treatment, consistent with existing treatment-related theoretical models that stress a need for initial prioritization of ED symptoms when initiating treatment [91–93]. Further, targeting ED symptoms in HLOCs via repeated mealtimes and exposure to previously avoided cues may, in fact, temporarily increase symptoms of anxiety and depression [94]. Overall, our findings underscore the ongoing need to better understand differential symptom trajectories in HLOCs and explore clinical-operational methods to improve outcomes among those currently reporting minimal change in symptoms.

Our second aim of the current investigation was to explore predictors of symptom change in our sample. Psychiatric comorbidity, ED diagnosis, and admission ED severity emerged as consistent predictors across different outcomes, although not always consistent in pattern or in the expected direction. Regarding ED diagnoses, those with BN, BED, and OSFED/UFED seemed to demonstrate greater improvement in cognitive and affective symptoms (EDE-Q global scores; depression; anxiety) than those with a diagnosis of AN, in a similar manner to past literature [20, 45, 95]. One possible interpretation of these results is that those with AN may simply be at higher risk for poorer treatment outcomes in HLOCs; however, this finding may be accounted for by group differences in self-reported symptom severity, as those with AN also endorsed lower EDE-Q global scores at admission. Indeed, *higher* admission EDE-Q global scores were associated with greater change in cognitive ED symptoms, depression, and anxiety, a finding that has some precedent in past literature [96]. It is possible that these findings are a result of regression to the mean or floor effects for those with lower symptoms at admission; on the other hand, those with elevated symptoms may present to treatment with greater impairment and thus motivation to change.

In addition to certain ED diagnoses and higher EDE-Q global scores, the AN-BP subtype in our sample was associated with greater improvement in EDE-Q global scores and depression and no differences in average weight-related trajectories. Although some past research has suggested that the binge/purge subtype is associated with worse outcomes [45, 97], findings are mixed and suggest more frequent association with treatment dropout, rather than symptom change. Notably, while those with AN-BP in our sample were slightly less likely than those with AN-R to have missing data at discharge, they were equally likely to have a routine discharge from treatment. Additionally, psychiatric comorbidity was associated with worse treatment response in models exploring change in EDE-Q global scores but better response in weight-focused models. The reason for this inconsistency is unclear; while psychiatric comorbidity has been highlighted as a potentially important prognostic indicator in past work [45, 97], there have been inconsistent findings in this area as well, likely secondary to variable operationalization of comorbidity (i.e., dichotomous outcome; diagnosis-specific variables) and outcomes. Consistent with past data, it could also be the case that those with comorbidity were more likely to be prescribed psychotropic medications [98, 99], some of which are associated with secondary weight gain [100]. Our data support the possibility that those with psychiatric comorbidities may demonstrate slower improvements in cognitive symptoms, perhaps secondary to greater complexity of clinical presentation and/or secondary symptoms interfering with ED-related treatment progress [34, 39, 45, 97], but suggest that progress in weight gain may remain intact. Further, younger age was associated with greater weight gain over time but was not a predictor of change in cognitive or affective outcomes. Prior work has suggested that younger age at presentation may be related to outcome given strong relationships between age and length of illness [34, 101–103]; our work further adds to this literature but highlights that this relation may be specific to weight, rather than cognitive ED symptoms. Direct tests of why younger age may relate to increased weight gain in AN are limited in the literature. Future work might consider testing the influence of psychological (e.g., greater propensity to follow program rules) or biological (e.g., pubertal processes) explanations.

Of note, few predictors emerged as significant in predicting change in binge eating and self-induced vomiting behaviors in our sample; there were no predictors of changes in binge eating in our sample, and only baseline ED severity predicted change in vomiting over time. Therefore, while our study is notable regarding increased representation of adolescents with BN and BED compared to past work, additional research—ideally with even larger sample sizes—is critical to identify predictors of outcome in these groups. Additionally, future work in this area might consider exploring distinct predictors of outcome that have been identified in past literature focused on binge-spectrum EDs, including emotion regulation difficulties, facets of impulsivity, reward sensitivity, cognitive flexibility, and obsessive-compulsive symptoms [104–109]. Overall, there is a critical need for additional work on predictors of outcome within treatment for adolescent EDs, including studies that explore and directly measure candidate processes through which each observed effect may operate (e.g., what processes account for observed effects indicating that ED severity predicts better outcome?).

Finally, post-hoc analyses exploring the effect of US region on outcomes suggested that those in Western-based treatment sites demonstrated slightly less change in symptoms over time, compared to those in Southern treatment centers. While it could be the case that these effects can be accounted for by differences in legal practices and cultural differences, it is also important to note that, compared to the South, treatment centers from the West were more frequently inpatient or residential and admitted patients with significantly higher admission EDE-Q, PHQ-9, and GAD-7 scores, as well as lower %EBWs, which may account for these findings. Future research that better accounts for potential differences in treatment center and geographical region will likely enhance the rigor of this line of work.

### Limitations

Like other existing naturalistic treatment research in HLOCs [20, 23, 28, 40, 110], our study had notable limitations. First, although our sample represents the largest adolescent-only sample in the existing literature on HLOCs and EDs and includes a range of different ED presentations, our participants were relatively homogeneous in gender, race, and ethnicity. While the relative representation of demographic groups was similar to other past literature [17, 20, 23, 55, 111], this limitation inhibits the generalizability of our findings to other groups and reinforces the critical need to characterize systems-level processes that affect differential rates of treatment presentation among individuals from diverse gender, racial, and ethnic identities [112–114]. Second, data were only collected from participants at three time points throughout treatment; because individuals entered and discharged at different levels of care, stepdown assessments were also conducted at different levels of care. These variations in assessment administration introduce heterogeneity into models. While we attempted to improve our approach by separating out those admitting to IP and RES from those admitting to PHP and IOP levels of care, this remains a noteworthy limitation. Additionally, no data were collected following discharge from treatment; lack of data on long-term outcomes following discharge from HLOCs is a significant limitation of our study and most existing work in the literature. Third, consistent with similar past studies on this topic [14, 20, 23, 55], there were high levels of missingness in our data at follow-up time points, although we attempted to minimize the bias of missingness through use of full-information maximum likelihood estimation. Notably, our missing data analyses suggested some systematic patterns in missingness, which may bias our results. Additionally, our sample included a small number of youth who were less than 11 years old (*n* = 12) or less than 13 years old (*n* = 178). While many of the self-report measurements have been used in older children/younger adolescent samples [62, 64, 115–120], there are less data regarding the validity of these measures for those age 11. We recommend that future work consider incorporation of developmentally-sensitive measures, such as versions of the EDE-Q that have been adapted specifically for youth [121]. Moreover, most collected data (except for weight) were self-report measures completed by adolescents, which may have resulted in bias due to underreporting and minimization [84]. While parents/caregivers were asked to complete ED-related symptom reports, response rates were low, rendering the data insufficient for analysis. This represents a considerable limitation of our study; future work should include collateral symptom reports completed by caregivers, other support people, or treatment staff, as research indicates that these reports can diverge meaningfully from youth’s self-report and provide critical information regarding ED symptoms [69, 84, 122, 123]. Finally, as we note above, our study design and lack of data on outpatient care within a matched sample did not permit evaluation of questions related to relative cost and effectiveness of HLOC compared to outpatient treatment, as well as questions related to clinical decision-making regarding referral and treatment recommendations in HLOC. While these questions may be challenging to test empirically, collecting these data will be essential for understanding the most effective ways to provide efficient and effective care for youth with EDs.

### Future directions & conclusions

In the current study, we described data from the largest study to date characterizing treatment outcomes in HLOCs for adolescent EDs. Overall, results indicated that adolescents demonstrated decreases in ED symptoms, anxiety, and depression over time, with the most consistent clinically significant benefit occurring for ED cognitive symptoms and weight, indeed the main indication for their admission. Predictor analyses suggested those with AN may demonstrate worse response compared to other ED diagnoses, and that symptom severity at admission, psychiatric comorbidity, and age may differentially relate to symptom progress depending on the domain in question. Future research must build upon these findings through more precisely testing the impact of different treatment components (e.g., group-based programming versus individual therapy) and medical comorbidities, evaluating long-term outcomes at follow-up, and expanding samples to include individuals with a broader range of diagnoses, comorbidities, and identities. Exploring which aspects of treatment may account for outcome can inform efforts to add or eliminate non-essential components, and research on predictors and the mechanisms of predictor effects can provide insights regarding the design of adjunctive interventions targeting those mechanisms. While naturalistic research in these treatment settings can be challenging to conduct and is plagued by numerous methodological limitations, these data offer important, “real world” information that can be used to inform the field’s understanding of HLOCs and shifts in treatment offerings designed to improve prognosis for this patient population.

## Electronic supplementary material

Below is the link to the electronic supplementary material.

Supplementary Material 1

## Data Availability

Data supporting the current manuscript are available from the senior authors upon reasonable request.
