# Supplementary material for: Predictors of treatment outcome in higher levels of care among a large sample of adolescents with heterogeneous eating disorders
Source: Child Adolesc Psychiatry Ment Health. 2024 Oct 17;18:131. doi: 10.1186/s13034-024-00819-8 (PMC11488273; doi:10.1186/s13034-024-00819-8)
Supplement: Supplementary file 1 — Supplementary Material 1 [file 13034_2024_819_MOESM1_ESM.docx]

**Supplemental Information: Predictors of Treatment Outcome in Higher Levels of Care Among a Large Sample of Adolescents with Heterogeneous Eating Disorders**

**Supplemental Methods**

**Missing Data**

Around 78% of the sample (n = 1,477) completed surveys at either step-down or discharge (i.e., either stepdown or follow-up). We used independent samples t-tests, chi-square analyses, and Fisher’s exact tests to probe differences between those with missing data at stepdown and/or discharge. Specifically, we explored whether differences in missingness related to age at admission, EDE-Q global scores, percent of expected body weight at admission, baseline binge eating episodes, self-induced vomiting, gender, race/ethnicity, ED diagnosis, psychiatric comorbidity, and reason for discharge (i.e., routine; non-routine; other).

**Stepdown Missingness.** Compared to those with complete data or those for whom stepdown self-reports were not expected (i.e., they did not step down during their treatment stay), those with true missingness at stepdown were younger (*M* = 14.58, *SD* = 1.70), compared to those with complete or no stepdown data (*M* = 14.89, *SD* = 1.62), *t*(1969) = 3.07, *p* = 002. There were no significant mean differences across groups in EDE-Q global scores, percent of expected body weight (%EBW), binge eating episodes, or vomiting episodes (*p*’s > .05). Chi-square and Fisher’s exact tests suggested that those with missing step-down data were more likely to be admitted to the IP level of care, *χ*(3) = 174.88, *p* < .001. There were no differences in gender, race/ethnicity, sex, ED diagnosis, psychiatric comorbidity, or reason for discharge across missingness groups.

**Discharge Missingness.** Those with missing data at discharge were more likely to have a higher %EBW at admission (*M* = 100.70; *SD* = 25.60) than those with complete data (*M* = 97.11; *SD* = 22.57), *t*(1938.35) = -3.29, *p* = .001. There were no significant differences across missingness group in age, EDE-Q global scores at admission, binge eating episodes, or self-induced vomiting. Those with missing data at discharge were more likely to have a non-routine discharge *χ*(2) = 89.31, *p* < .001, likely secondary to logistical challenges in securing self-report questionnaires with unexpected discharges. There were significant gender differences in missingness, such that cisgender females were less likely to have missing data than other gender groups, *χ*(5) = 15.81, *p* = .007. Race significantly related to missingness, *χ*(8) = 23.75, *p* = .003, such that participants identifying at Hispanic/Latinx were more likely to have missing data at discharge, and White participants were less likely to have missing data compared to other groups.

There were also significant diagnostic differences in missingness, *χ*(4) = 20.75, *p* < .001, some of which could be accounted for by diagnostic differences in routine versus non-routine discharges. Post-hoc tests indicated that those with BN, and in some instances, OSFED, were more likely to have missing data at stepdown and/or discharge compared to those with AN-R and AN-BP, secondary to having fewer step-downs and more frequent non-routine discharges. Direct comparisons between AN subtypes indicated that those with AN-BP were slightly more likely to have missing data at discharge than those with AN-R, *χ*(4) = 4.08, *p* = .043, but more likely to have complete data than those with BN, *χ*(1) = 5.87, *p* = .015, and did not differ in the rate of non-routine discharges, *χ*(2) = 4.76, *p* = .092. Those without psychiatric comorbidities were more likely to have missing data, *χ*(1) = 8.35, *p* = .004. Finally, level of care at entry related to discharge missingness, *χ*(2) = 89.31, *p* < .001, with those entering at PHP or IOP more likely to have missing data. Notably, variables that were related to missingness had a significant degree of overlap, rendering it challenging to interpret patterns in missingness.

Table S1.

*Estimates of fixed effects from predictor models, controlling for geographical region and level of care* (full sample)

|  |  | **EDE-Q Model** | | | | |  | **PHQ-9 Model** | | | | |  | **GAD Model** | | | | |
| --- | --- | --- | --- | --- | --- | --- | --- | --- | --- | --- | --- | --- | --- | --- | --- | --- | --- | --- |
| Predictor |  | Est. | *SE* | *p* | *R_p_^2^* | *95%CI* |  | Est. | *SE* | *p* | *R_p_^2^* | *95%CI* |  | Est. | *SE* | *p* | *R_p_^2^* | *95%CI* |
| Intercept |  | **3.36** | **0.07** | **<.001** | **.65** | **.63, .66** |  | **12.25** | **0.41** | **<.001** | **.35** | **.33, .38** |  | **11.41** | **0.42** | **<.001** | **.26** | **.24, .30** |
| Time |  | **-0.02** | **0.00** | **<.001** | **.03** | **.02, .04** |  | **-0.05** | **0.01** | **<.001** | **.01** | **.00, .02** |  | **-0.03** | **0.01** | **<.001** | **.01** | **00, .01** |
| EDEQ Admit |  | **0.89** | **0.01** | **<.001** | **.56** | **.54, .58** |  | **2.63** | **0.82** | **<.001** | **.25** | **.22, .27** |  | **1.81** | **0.08** | **<.001** | **.17** | **.14, .19** |
| Age |  | 0.01 | 0.01 | .635 | .00 | .00, .00 |  | **0.30** | **0.08** | **<.001** | **.00** | **.00, .01** |  | 0.09 | 0.09 | .310 | .00 | .00, .00 |
| LOS |  | **0.00** | **0.00** | **<.001** | **.02** | **.01, .03** |  | **0.01** | **0.00** | **.002** | **.00** | **.00, .01** |  | **0.01** | **0.00** | **.001** | **.01** | **.00, .01** |
| AN-BP^1^ |  | -0.03 | 0.06 | .604 | .00 | .00, .00 |  | 0.35 | 0.40 | .380 | .00 | .00, .00 |  | -0.24 | 0.41 | .562 | .00 | .00, .00 |
| Comorbid |  | -0.15 | 0.05 | .011 | **.00** | **.00, .01** |  | **1.20** | **0.36** | **.380** | **.00** | **.00, .01** |  | 1.00 | 0.37 | .006 | .00 | .00, .01 |
| BN^2^ |  | 0.12 | 0.10 | .220 | .00 | .00, .00 |  | 1.06 | 0.60 | .079 | .00 | .00, .00 |  | -0.15 | 0.61 | .811 | .00 | .00, .00 |
| BED^2^ |  | -0.08 | 0.16 | .633 | .00 | .00, .00 |  | 0.81 | 0.99 | .413 | .00 | .00, .00 |  | 0.05 | 1.15 | .965 | .00 | .00, .00 |
| OSFED/UF^2^ |  | 0.03 | 0.05 | .498 | .00 | .00, .00 |  | 0.74 | 0.31 | .016 | .00 | .00, .01 |  | -0.06 | 0.33 | .847 | .00 | .00, .00 |
| West^3^ |  | 0.14 | 0.05 | .006 | .00 | .00, .01 |  | 0.80 | 0.32 | .012 | .00 | .00, .01 |  | 0.73 | 0.33 | .028 | .00 | .00, .01 |
| Midwest^3^ |  | 0.07 | 0.05 | .163 | .00 | .00, .00 |  | 0.04 | 0.33 | .893 | .00 | .00, .00 |  | 0.37 | 0.35 | .290 | .00 | .00, .00 |
| Northeast^3^ |  | -0.06 | 0.29 | .840 | .00 | .00, .00 |  | -1.69 | 1.76 | .339 | .00 | .00, .00 |  | -2.80 | 2.99 | .349 | .00 | .00, .00 |
| LOC |  | **0.15** | **0.05** | **<.001** | **.00** | **.00, .01** |  | 0.52 | 0.33 | .893 | .00 | .00, .00 |  | 0.32 | 0.30 | .281 | .00 | .00, .00 |
| Time*Age |  | -0.00 | 0.00 | .648 | .00 | .00, .00 |  | -0.00 | 0.00 | .160 | .00 | .00, .00 |  | -0.00 | 0.00 | .290 | .00 | .00, .00 |
| Time*AN-BP^1^ |  | **-0.01** | **0.00** | **<.001** | **.00** | **.00, .01** |  | -0.01 | 0.01 | .110 | .00 | .00, .00 |  | -0.01 | 0.01 | .132 | .00 | .00, .00 |
| Time*Comorbid |  | **0.01** | **0.00** | **<.001** | **.00** | **.00, .01** |  | 0.00 | 0.01 | .612 | .00 | .00, .00 |  | 0.01 | 0.01 | .475 | .00 | .00, .00 |
| Time*BN^2^ |  | **-0.02** | **0.00** | **<.001** | **.01** | **.01, .03** |  | **-0.08** | **0.02** | **<.001** | **.01** | **.00, .01** |  | **-0.05** | **0.01** | **.002** | **.00** | **.00, .01** |
| Time*BED^2^ |  | -0.01 | 0.01 | .032 | .00 | .00, .01 |  | -0.04 | 0.02 | .159 | .00 | .00, .00 |  | -0.03 | 0.03 | .242 | .00 | .00, .00 |
| Time*OSFED^2^ |  | **-0.01** | **0.00** | **<.001** | **.01** | **.00, .02** |  | -0.01 | 0.01 | .007 | .00 | .00, .01 |  | -0.01 | 0.01 | .185 | .00 | .00, .00 |
| Time*EDEQ Ad. |  | **0.01** | **0.00** | **<.001** | **.07** | **.06, .09** |  | **-0.02** | **0.00** | **<.001** | **.02** | **.01, .03** |  | -0.00 | 0.00 | .004 | .00 | .00, .01 |
| Time*West^3^ |  | **0.00** | **0.00** | **<.001** | **.00** | **.00, .01** |  | **0.03** | **0.01** | **<.001** | **.01** | **.00, .01** |  | **0.02** | **0.01** | **<.001** | **.01** | **00, .01** |
| Time*Midwest^3^ |  | 0.00 | 0.00 | .786 | .00 | .00, .00 |  | 0.00 | 0.01 | .975 | .00 | .00, .00 |  | -0.00 | 0.01 | .871 | .00 | .00, .00 |
| Time*Northeast^3^ |  | 0.02 | 0.01 | .014 | .00 | .00, .01 |  | 0.04 | 0.03 | .274 | .00 | .00, .00 |  | 0.04 | 0.12 | .738 | .00 | .00, .00 |
| Time*LOC |  | **-0.00** | **0.00** | **<.001** | **.00** | **.00, .01** |  | -0.02 | 0.01 | .004 | .00 | .00, .01 |  | -0.01 | 0.01 | .027 | .00 | .00, .00 |

*Note.* LOS = length of stay; AN-BP = binge/purge subtype of AN; BN = bulimia nervosa; BED = binge eating disorder; OSFED = other specified ED/unspecified ED, EDE-Q Ad. = EDE-Q Global score at baseline/admission; LOC = level of care; effects with a *p*-value < .003 are bolded.

^1^This effect refers to the impact of having an AN-BP diagnosis on the outcome, compared to having all other diagnoses

^2^Diagnosis-specific effects indicate the impact of having the listed diagnosis on the outcome, compared to those with AN (reference group)

^3^The effect refers to the impact of being enrolled in a treatment center within a given US region, compared to those enrolled in treatment centers in the Southern US

| Table S2.  *Estimates of fixed effects from predictor models for %EBW* | | | |
| --- | --- | --- | --- |
| Predictor | Est. | *SE* | *p* |
| **Intercept** | **84.50** | **0.89** | **<.001** |
| **Time** | **0.20** | **0.01** | **<.001** |
| **EDEQ Admission** | **0.74** | **0.18** | **<.001** |
| **Age** | **-1.56** | **0.18** | **<.001** |
| **LOS** | **-0.52** | **0.01** | **<.001** |
| **AN-BP^1^** | **4.65** | **0.70** | **<.001** |
| Comorbid | 0.64 | 0.79 | .417 |
| **LOC** | **4.36** | **0.65** | **<.001** |
| Northeast^2^ | -0.18 | 4.93 | .972 |
| West^2^ | 1.20 | 0.70 | .085 |
| Midwest^2^ | 0.58 | 0.77 | .452 |
| Time*Age | -0.01 | 0.00 | .017 |
| Time*AN-BP^1^ | -0.01 | 0.01 | .279 |
| Time*Comorbid | 0.01 | 0.01 | .310 |
| **Time*EDEQ Admission** | **-0.01** | **0.00** | **<.001** |
| **Time*LOC** | **-0.07** | **0.01** | **<.001** |
| Time*Northeast^2^ | -0.03 | 0.06 | .507 |
| Time*West^2^ | 0.02 | 0.01 | .016 |
| Time*Midwest^2^ | 0.01 | 0.01 | .150 |
| *Note.* LOS = length of stay; AN-BP = anorexia nervosa, binge/purge subtype; EDEQ Admission = Eating Disorder Examination-Questionnaire Global score at baseline; LOC = level of care. Effects with a p-value < .003 are bolded.  ^1^This effect refers to the impact of having an AN-BP diagnosis on body weight, compared to AN-R  ^2^The effect refers to the impact of being enrolled in a treatment center within a given US region, compared to those enrolled in treatment centers in the Southern US | | | |

| Table S3.  *Estimates of fixed effects from predictor models for binge eating and self-induced vomiting* | | | | | | | | |
| --- | --- | --- | --- | --- | --- | --- | --- | --- |
|  |  | **Binge Eating Model** (*n* = 405) | | |  | **Vomiting Model** (*n* = 414) | | |
| Predictor |  | Est. | *SE* | *p* |  | Est. | *SE* | *p* |
| Intercept |  | 0.55 | 0.38 | .149 |  | **2.67** | **0.35** | **<.001** |
| Time |  | **-0.06** | **0.01** | **<.001** |  | **-0.16** | **0.02** | **<.001** |
| EDEQ Admission |  | **0.26** | **0.08** | **.001** |  | **0.28** | **0.07** | **<.001** |
| Age |  | 0.07 | 0.08 | .348 |  | 0.07 | 0.07 | .350 |
| LOS |  | 0.00 | 0.00 | .126 |  | 0.00 | 0.00 | .037 |
| AN-BP^1^ |  | -- | -- | -- |  | -0.35 | 0.23 | .133 |
| Comorbid |  | 0.02 | 0.32 | .942 |  | -0.05 | 0.29 | .865 |
| BN^2^ |  | **1.12** | **0.28** | **<.001** |  | -- | -- | -- |
| BED^2^ |  | **2.05** | **0.42** | **<.001** |  | -- | -- | -- |
| LOC |  | 0.13 | 0.25 | .607 |  | -0.36 | 0.22 | .101 |
| West^3^ |  | 0.58 | 0.29 | .045 |  | -0.11 | 0.25 | .650 |
| Midwest^3^ |  | 0.17 | 0.28 | .547 |  | -0.09 | 0.25 | .731 |
| Northeast^3*^ |  | -0.10 | 2.17 | .962 |  | -- | -- | -- |
| Time*Age |  | 0.00 | 0.00 | .532 |  | 0.00 | 0.00 | .353 |
| Time*AN-BP^1^ |  | -- | -- | -- |  | **0.03** | **0.01** | **<.001** |
| Time*Comorbid |  | 0.01 | 0.01 | .532 |  | **0.04** | **0.01** | **<.001** |
| Time*BN^1^ |  | 0.00 | 0.01 | .870 |  | -- | -- | -- |
| Time*BED^1^ |  | -0.02 | 0.02 | .191 |  | -- | -- | -- |
| Time*EDEQ Admission |  | 0.00 | 0.00 | .356 |  | **0.01** | **0.00** | **.005** |
| Time*LOC |  | 0.02 | 0.01 | .023 |  | **0.05** | **0.01** | **<.001** |
| Time*West^3^ |  | 0.02 | 0.01 | .017 |  | **0.05** | **0.01** | **<.001** |
| Time*Midwest^3^ |  | 0.00 | 0.01 | .706 |  | 0.03 | 0.01 | .003 |
| Time*Northeast^3*^ |  | -- | -- | -- |  | -- | -- | -- |
| *Note.* LOS = length of stay; AN-BP = anorexia nervosa, binge/purge subtype; BN = bulimia nervosa; BED = binge eating disorder; EDEQ Admission= Eating Disorder Examination-Questionnaire Global score at baseline/admission; LOC = level of care; effects with a *p*-value < .003 are bolded.  ^1^This effect refers to the impact of having an AN-BP diagnosis on the outcome, compared to having BN  ^2^ Diagnosis-specific effects indicate the impact of having the listed diagnosis on the outcome, compared to those with AN-BP (reference group)  ^3^The effect refers to the impact of being enrolled in a treatment center within a given US region, compared to those enrolled in treatment centers in the Southern US  ^*^This effect was removed from the model due to having insufficient cell sizes to explore the interaction | | | | | | | | |
